# Supplementary Report of Ligation of External Iliac for Aneurism of Femoral Artery

**Published:** 1887-05

**Authors:** J. McF. Gaston

**Affiliations:** Professor of Surgery in the Southern Medical College, Atlanta, Ga.


### SUPPLEMENTARY REPORT OF LIGATION OF EXTERNAL ILIAC FOR ANEURISM OF FEMORAL ARTERY.

By J. McF. GASTON, M. D.,
Professor of Surgery in the Southern Medical College, Atlanta, Ga.

In the March number of the RECORD there appeared a notice of my operation, on the 30th of January, for a femoral aneurism which co-existed with a popliteal aneurism, relieved by ligation of the femoral below Scarpa's triangle two months previously.

On the 18th of February, the silk ligature being still retained, an elastic strap was secured to it and fastened to a be't around the waist of the patient. A small roll of cotton was placed near the upper angle of the incision, over which the loose end of the ligature passed, so as to give a perpendicular traction upon the confined loop. Some additional force, by drawing slightly with the hand daily, caused tension which assisted in the dislodging of the ligature, so that the repetition of this on the morning of the 24th brought it away. On the same day the patient removed the ligature from the wound connected with the femoral, where it had remained for three months, being cut off close at the time of the operation. There was more suppuration from this closed incision treated upon antiseptic principles than from the open wound after ligation of the external iliac, and this certainly offers no encouragement to expect absorption or encysting of silk ligatures upon arteries in the thigh, whatever may be the result in the pelvic viscera.

A very interesting sequel of the ligation of the external iliac was the cessation of pulsation in the tumor, with its gradual diminution in size and firmness, for six weeks, when pulsation was perceptible in the small mass that remained, and has continued up to the present time. Having watched the case closely for ten consecutive days without observing any increase in the tumor or the pulsation, I suspected that it might result from the redevelopment of the profunda through the anastomosing vessels, and proceeded to make an examination by lifting up the tumor with my fingers so as to relieve the impulse from beneath. Much to my relief no pulsation could any longer be felt in the mass, though the evidence of pulsation beneath, in the line of the profunda, was evident. Thus the embarrassment of a supposed reproduction of the aneurism is at rest, and my double operation turns out to be entirely successful in curing both the popliteal and femoral aneurisms, with a restoration of the circulation to the limb.

